# Fatal Basilar Thrombosis Possibly Related to Minor Cervical Trauma: A Case Report

**DOI:** 10.1155/2010/401978

**Published:** 2010-08-08

**Authors:** Elmaz Shaqiri, Gentian Vyshka, Admir Sinamati, Besim Ymaj, Zija Ismaili

**Affiliations:** ^1^Institute of Legal Medicine, University Hospital Center “Mother Theresa”, Rr. Dibres 371, 1005 Tirana, Albania; ^2^Service of Neurology, University Hospital Center “Mother Theresa”, Rr. Dibres 371, 1005 Tirana, Albania

## Abstract

*Background*. Basilar thrombosis is a potentially fatal event, whose traumatic etiology has been repeatedly stated. *Methods*. We performed the autopsy and the microscopic examination of the brain stem structures from an individual, whose sudden death raised logical suspicions regarding the causative factor. *Results*. The brain was swollen and clearly hyperemic; a massive basilar thrombus with complete occlusion of the median segment of the basilar artery was macroscopically seen. The brainstem showed an infarcted zone in the pons, with secondary hemorrhagic changes, mainly in the form of multiple petechial hemorrhages. Pontine arteries showed extensive alterations, mainly in the form of severed endothelium, which suggested a thrombotic-traumatic mechanism as the main etiological factor. *Conclusions*. Minor injuries, such as slight whiplashes, abrupt neck movements, neck trauma related to the slowing down of the vehicles, and critical neck positioning, can all of them explain a thrombotic event in the basilar artery, leading to a fatal occurrence. Other risk factors may obviously concur, but their importance seems unclear.

## 1. Case Report

A 53-year-old man was found dead from the driver of a passenger van, upon arrival at the city of destination. With other passengers leaving the van, he waited for the man to descend, thinking that he was asleep. The victim was immediately brought at the city hospital, where the death was declared. The autopsy was performed immediately the same day; samples were sent at the Institute of Forensic Medicine where microscopy was made on the organs of interest. 

The medical staff examined the previous medical files and documentation of the victim; no chronic diseases were suggested. He was referred to be a heavy smoker (30 years with twenty cigarettes per day); he was obese; but no hypertension or other major risk factors were found. Toxicology revealed no signs or suggestions for medications, intoxications, or recent use of ethanol. 

The day of the trip the victim woke up early and left his home at dawn; he used to go working every day at the city of destination. 

The forensic pathologist performing the autopsy found nothing unusual at the thoracic cavity, no enlargement of cardiac rooms was seen. The autopsy showed no thrombi inside the cardiac cavities; nor was any sign of thrombotic event found in the major extra cranial vessels. The coronary arteries showed atherotic signs compatible with the age and the body mass (his weight was 97 kilograms for a height of 165 centimeters). 

**Figure 1:**
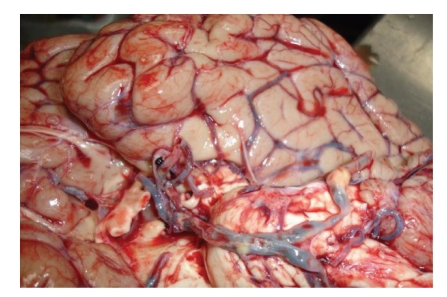
Macroscopy of the brain; the occluded thrombotic basilar artery is seen.

The brain was swollen and clearly hyperemic; a massive basilar thrombus with complete occlusion of the median segment of the basilar artery was macroscopically seen (Figure 1).

**Figure 2:**
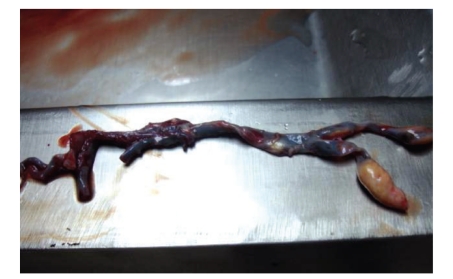
The occlusion of the basilar artery involved mainly the median portion. Note the atheromatous basilar artery, with an extensive and fresh thrombus.

The dissected basilar artery, from the origin of the vertebral arteries to its top, is shown (Figure 2); samples of brain stem tissues were microscopically examined. The occlusion of the basilar artery was in the median portion; thrombotic fragments were found as well in the oral portion of the basilar artery. 

**Figure 3:**
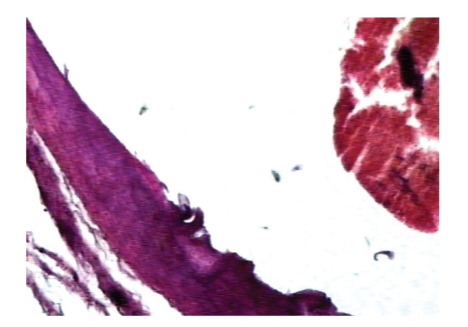
Severed endothelium in the territory of a pontine artery (center of the figure) with fragments of a fresh thrombus (right in the figure).

The brainstem showed an infarcted zone in the pons, with secondary hemorrhagic changes, mainly in the form of multiple petechial hemorrhages. Pontine arteries showed extensive alterations, mainly in the form of severed endothelium, which suggested a thrombotic-traumatic mechanism as the main etiological factor (Figure 3). 

**Figure 4:**
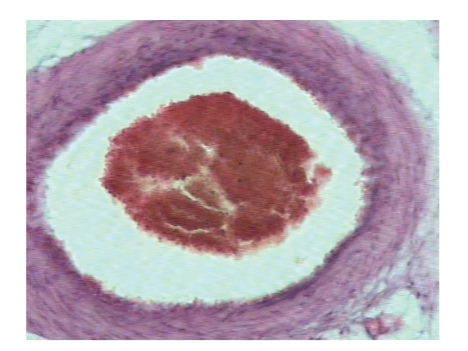
Intraluminal thrombus.

**Figure 5:**
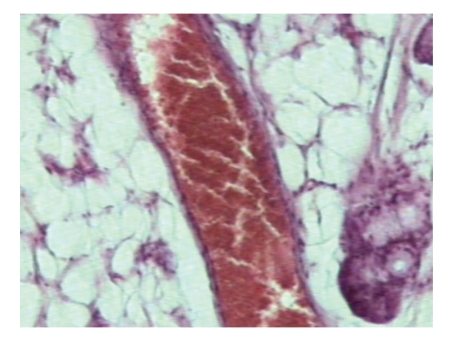
A thrombosed arteriole in the sagittal section. The fresh thrombus expanded longitudinally. There is evidence of adjacent cellular edema.

Microscopically, evidence of thrombotic fragments in the pontine arteries was found as well (Figure 4), with thrombus formation expanding longitudinally (Figure 5).

The occlusion of the basilar artery was considered from the forensic experts and the consultant staff as the causative factor of death. In face of lacking other etiological factors that could have explained the impressive thrombotic occlusion of basilar artery, leading to immediate death, the experts concluded that minor cervical trauma could be responsible. In fact, the victim was found seated in the van in a very precarious position in the last row. Interviewed from the coroner, the passengers referred a very unstable trip in a bumpy and poorly driven car, with the driver obliged to slow down several times, causing even some displacement of the passengers inside the vehicle. The shocks that the victim absorbed on the posterior region of his neck during the braking episodes possibly caused the thrombotic occlusion of the basilar artery, with expansion of the occluding fragments to the pontine arteries; the immediate death probably followed one of those shocking moments, due to pontine infarction. The role of other causative or predisposing factors remained unclear. 

## 2. Discussion

The thrombotic occlusion of the basilar artery is almost fatal, and all pioneer studies have been convergent on the issue [1, 2]. The probability that the basilar thrombosis might be the cause of a sudden death has been described in the literature since 1868 [3]. However, a fatal basilar thrombosis related only with a minor trauma, to our knowledge, has been described and published only more than a century later, in 1990 [4]. The authors referring such a case accept as well that no signs of external trauma in the cervical spine, soft tissues of the neck, or adjacent structures were found, albeit the occlusion of the basilar artery was obvious and interested the entire length of the artery [4]. The possibility that an occlusion of the basilar artery might follow even a minor whiplash injury has been emphasized as well; the thrombotic event might follow the trauma in a time delay that might reach several months [5]. Other authors have described even a group of patients in whom vertebrobasilar ischemia came on after the extension of their necks over the edge of a hairdresser's sink, while having their hair shampooed [6]. Such a strange and unusual presentation of a so-called “beauty parlor syndrome”, where the trauma to the neck has of course to be minor and irrelevant, if inexistent, has been also reported from other sources [7]. The same author that coined the above-mentioned term of the syndrome emphasizes that the critical neck position might be an independent risk factor for a posterior circulation stroke [8]. 

While previously the chiropractic manipulation was considered to be an important factor leading to traumatic vertebrobasilar ischemia, recent data suggest the motor vehicle accidents to be the most common cause of traumatic vertebrobasilar ischemia, irrespective to the severity of the accident [9]. Through suggesting the trauma as a causative factor, almost everywhere in the literature this trauma of the cervical region is implied as a blunt one [10]. 

In discussing the mechanism of injury, some authors speculate that hyperflexion of the neck is the dominant mechanism of injury [11]. A list of risk factors has been suggested, with obesity and cigarette smoking being among them [12]. In our case, there was no external sign of traumatic shock to the cervical region, nor any internal hematoma following the plane dissection during the autopsy. Anyway, other factors such as obesity and/or cigarette smoking might have been playing a concurrent role. 

The case described in our paper was abruptly fatal; however the prognosis of basilar artery occlusion even in the cases of a more prolonged time course is generally poor. The authors mention several factors that might influence the immediate and long-term outcome, such as the decreased level of consciousness, the presence of dysarthria, the pupillary abnormalities, bulbar symptoms, bilateral involvement of cerebellar hemispheres, tetraplegy, and even the cardiac cause of embolism [13]. All patients with such factors referred to by this study either have died or suffered from severe further disability [13]. 

Basilar artery occlusion may present without significant warning symptoms, or with fluctuating symptoms progressing to severe disability and death; the latter might have a similar course with that of a brainstem tumor, leading as well to a sudden, immediate death [14, 15]. The transient symptoms that precede the major event of a basilar thrombosis include visual spots, blurred vision, diplopia, facial paresthesias, vertigo, ataxia, dysarthria, and transient hemiparesis or tetraparesis. Based on the temporal profile of the symptomatology, the basilar thrombosis will manifest in several clinical pictures, such as

- sudden onset of symptomatology, with bulbar signs, impaired consciousness, probably progressing to a locked-in syndrome and coma; potentially such cases can be immediately fatal;
- a more gradual course of the above-mentioned symptomatology, leading later to the full and final clinical picture of a tetraplegic, comatose patient;
- prodromal symptoms, mostly transient in their nature, such as vision impairment, convulsive-like jerking, and hemiparesis, preceding the total occlusion by days or weeks.

In nonfatal cases, the recanalization is considered an important requisite for a good functional outcome [16]. Although recanalization is achieved more frequently with intra-arterial thrombolysis, the morbidity and mortality of patients treated with such a procedure was not significantly different from the effect of intravenous thrombolysis, according to a study [17]. Tissue plasminogen activator (rtPA, recombinant) is the only pharmaceutical agent approved from FDA for the treatment of the acute ischemic stroke. On the other hand, more conventional therapies (anticoagulation, antiaggregants) offer only an insufficient efficacy, with a case fatality of 40%, and with 65% of survivors remaining dependent thereafter [18].
